# Unraveling syndrome-driven osteosarcoma: genetic insights and therapeutic frontiers

**DOI:** 10.1186/s13023-026-04243-3

**Published:** 2026-02-06

**Authors:** Yang Zhou, Wen Wang, Jin Qiu, Jingyang Huang, Laihua Fu, Songfeng Xu

**Affiliations:** 1 Department of Bone and Soft Tissue Oncology, National Cancer Center/National Clinical Research Center for Cancer/Cancer Hospital & Shenzhen Hospital, Chinese Academy of Medical Sciences and Peking Union Medical College, Shenzhen, 518116 China; 2 Department of Pathology, National Cancer Center/National Clinical Research Center for Cancer/Cancer Hospital & Shenzhen Hospital, Chinese Academy of Medical Sciences and Peking Union Medical College, Shenzhen, 518116 China

**Keywords:** Osteosarcoma, Hereditary syndromes, Clinical characteristics, Molecular mechanisms

## Abstract

Osteosarcoma is a highly malignant bone tumor, and a subset of cases is closely associated with hereditary syndromes. These syndrome-related osteosarcomas exhibit unique clinical features, molecular mechanisms, and therapeutic challenges. This review summarizes the current understanding of specific types of syndrome-related osteosarcomas, including those associated with Rothmund-Thomson syndrome, Li-Fraumeni syndrome, secondary osteosarcoma in retinoblastoma survivors, Werner syndrome, and Bloom syndrome. These syndromes are typically characterized by specific gene mutations or chromosomal instability, significantly increasing the risk of osteosarcoma development. However, the rarity and heterogeneity of syndrome-related osteosarcomas pose significant challenges for diagnosis and treatment, including difficulties in early detection, incomplete elucidation of molecular mechanisms, and limitations of conventional therapeutic approaches. This article aims to systematically review the clinical characteristics, molecular mechanisms, and therapeutic challenges of these syndromes, providing a comprehensive reference for clinicians and directions for future research.

## Introduction

Osteosarcoma, the most common primary malignant bone tumor in children and adolescents, is closely associated with various hereditary syndromes in terms of its pathogenesis. Studies indicate that approximately 28% of osteosarcoma patients carry highly penetrant pathogenic or likely pathogenic germline mutations, with TP53 mutations leading to Li-Fraumeni syndrome being the most prevalent, accounting for a significant proportion of syndrome-related osteosarcomas [1]. Additionally, hereditary retinoblastoma syndrome, Werner syndrome, Bloom syndrome, and Diamond-Blackfan anemia are also linked to osteosarcoma susceptibility [2]. These syndrome-related osteosarcomas often exhibit distinct clinical features, such as earlier onset in Li-Fraumeni syndrome -related cases (median diagnosis age of 16 years) and a higher incidence of secondary malignancies (e.g., breast cancer, soft tissue sarcomas) [3].

At the molecular level, driver mutations in syndrome-related osteosarcomas involve multiple signaling pathways. TP53 mutations lead to increased genomic instability, while RB1 loss promotes tumorigenesis through dysregulation of the cell cycle [4]. Mutations in DNA helicase genes, such as RECQL4, impair DNA damage repair, exacerbating genomic aberrations [5]. Furthermore, non-coding RNAs (e.g., miR-21, miR-34a) contribute to tumor progression by regulating MAPK and PI3K/Akt pathways [6]. Notably, CD133+ tumor stem cells in syndrome-related osteosarcomas exhibit enhanced metastatic and drug-resistant properties, potentially linked to Wnt/β-catenin pathway activation [7].

In addition to large-scale chromosomal rearrangements, osteosarcoma exhibits another hallmark of genomic instability at the sub-chromosomal level: a profound dependency on the Alternative Lengthening of Telomeres (ALT) mechanism for telomere maintenance. Unlike the majority of cancers which reactivate telomerase, osteosarcoma is one of the few tumor types where ALT is prevalently activated [8]. This reliance is mechanistically linked to the dysfunction of RecQ-family DNA helicases, which are crucial regulators of telomere homeostasis and homologous recombination. Specifically, the helicases RECQL4, WRN, and BLM play non-redundant roles in suppressing aberrant recombination at telomeres and resolving ALT-associated DNA structures, such as telomeric D-loops and stalled replication forks [9, 10].

The critical in vivo evidence for this link stems from the dramatically elevated osteosarcoma susceptibility observed in patients with germline RecQ helicase deficiency syndromes: Rothmund-Thomson, Werner, and Bloom [11]. The prevailing model posits that loss of functional RECQL4, WRN, or BLM leads to the accumulation of unresolved, replication-stressed telomeric DNA, which in turn acts as a substrate for aberrant homologous recombination. This creates a permissive genomic environment that precipitates ALT activation [10]. Consequently, in sporadic osteosarcomas, somatic alterations or dysregulation of these helicases may serve as a key permissive event, enabling cancer cells to exploit the ALT pathway to achieve replicative immortality. This ALT dependency, coupled with catastrophic chromosomal instability, defines the unique genomic landscape of osteosarcoma and presents a potential therapeutic vulnerability.

Therapeutically, syndrome-related osteosarcomas present multiple challenges. Conventional chemotherapy regimens (e.g., doxorubicin combined with cisplatin) have limited efficacy due to variations in patients’ sensitivity to DNA-damaging agents [12]. For instance, LFS patients face a significantly elevated risk of secondary tumors following radiotherapy or alkylating agent-based chemotherapy [13]. In recent years, targeted therapies (e.g., cabozantinib, sorafenib) and immune checkpoint inhibitors (e.g., pembrolizumab) have shown promise in specific subgroups, but their application requires individualized selection based on germline mutation profiles [14]. Additionally, drug development targeting molecules such as RECQL4 offers new avenues for future treatments.

In summary, the unique genetic background and clinical complexity of syndrome-related osteosarcomas necessitate multidisciplinary collaboration, integrating genetic testing, risk stratification, and personalized treatment strategies to improve patient outcomes. Future research should focus on elucidating the molecular mechanisms of driver mutations and their interactions with the tumor microenvironment, as well as exploring precise interventions for genetic predispositions. This review is the first to compare osteosarcomas associated with five rare syndromes, integrate the latest advancements in targeted therapy, and emphasize common molecular pathways, which is different from previous reviews that focused on a single syndrome.

## Syndromes and osteosarcoma

### Rothmund-Thomson syndrome and osteosarcoma

#### Clinical characteristics (Table 1)

**Table 1:** Clinical characteristics of syndrome-related osteosarcoma

Syndrome	Gene Mutation	Age of Onset	Tumor Location	Key Clinical Features
Rothmund-Thomson Syndrome	RECQL4	6–10 years	Multifocal or bilateral	Poikiloderma, growth retardation, cataracts [References 16, 17, 19]
Li-Fraumeni Syndrome	TP53	Median 16 years	Long bones	Early onset, multiple malignancies (e.g., breast cancer, brain tumors) [References 3, 3]
Retinoblastoma	RB1	10–20 years	Craniofacial or long bones	Radiation-induced, aggressive growth [References 20, 21]
Werner Syndrome	WRN	30–50 years	Pelvis, spine	Premature aging (cataracts, skin sclerosis), late onset [References 22, 23]
Bloom Syndrome	BLM	Adolescence	Long bones	Short stature, photosensitive rashes, immunodeficiency [References 1, 24]

Rothmund-Thomson syndrome (RTS) is a rare autosomal recessive genetic disorder characterized by poikiloderma, skeletal abnormalities, and cancer predisposition, particularly to osteosarcoma (OS). However, the risk of osteosarcoma is almost exclusively associated with Type II RTS (RECQL4 mutations, skeletal defects), whereas Type I carries a low risk. Clinical observations indicate that RTS patients typically develop erythema and photosensitivity in childhood, progressing to characteristic reticulated pigmentation and atrophy (poikiloderma) [15]. Approximately 30% of RTS patients develop osteosarcoma, with an earlier onset compared to sporadic cases (average diagnosis age of 6–10 years) [16]. Notably, RTS-related osteosarcomas often present as multifocal or bilateral, with a higher risk of recurrence [17]. Case reports indicate that some patients develop a second primary osteosarcoma in the contralateral limb within a decade of curing the initial tumor, underscoring the importance of long-term monitoring [18]. Additional systemic manifestations, such as growth retardation, cataracts, and sparse hair, aid in the early identification of high-risk individuals [19].

#### RECQL4 gene mutations and pathogenesis (Table 2)

**Table 2:** Germline mutations associated with Syndrome-Related Osteosarcoma

Syndrome	Mutated Gene	Protein Function	Risk of OS in syndrome carriers	Key References
Rothmund-Thomson Syndrome	RECQL4	DNA helicase, repair	30%	[11, 16, 25, 27]
Li-Fraumeni Syndrome	TP53	Tumor suppression, genomic stability	38%	[1, 31–33]
Retinoblastoma	RB1	Cell cycle regulation	36.2%	[34–36]
Werner Syndrome	WRN	DNA helicase, telomere maintenance	8%	[23, 37–39]
Bloom Syndrome	BLM	DNA helicase, homologous recombination	＜2%	[40–43]

The molecular basis of RTS is primarily attributed to biallelic pathogenic mutations in the RECQL4 gene [25]. RECQL4 encodes a DNA helicase involved in DNA replication, repair, and telomere maintenance [26]. Functional studies demonstrate that RECQL4 loss leads to genomic instability, including chromosomal breaks and copy number variations, thereby promoting tumorigenesis [27]. In RTS-related osteosarcomas, RECQL4 mutations often result in complete loss of protein function, leading to defects in cell cycle regulation (e.g., G1/S checkpoint deficiencies) and resistance to apoptosis [28]. Additionally, somatic TP53 mutations frequently coexist in RTS-related osteosarcomas, suggesting synergistic roles of RECQL4 and p53 pathways in tumor development [29]. Animal models further confirm that RECQL4-deficient mice are prone to osteosarcoma, with tumor tissues exhibiting aberrant Wnt/β-catenin pathway activation [30]. These findings provide a molecular basis for the high osteosarcoma susceptibility in RTS patients.

#### Treatment and prognosis (Table 3)

**Table 3:** Treatment challenges and emerging therapeutic options

Syndrome	Standard Drug Therapy	Challenges	Emerging therapeutic Options	References
Rothmund-Thomson Syndrome	Doxorubicin + Cisplatin	High chemotherapy toxicity, myelosuppression	Cabozantinib, Pazopanib	[15, 44, 45]
Li-Fraumeni Syndrome	Anthracyclines + Cisplatin	Concurrent malignancies	TP53 activators, Cabozantinib	[13, 47]
Retinoblastoma	Anthracyclines + Cisplatin	Risk of repeated radiation exposure	Local therapy, Immunotherapy	[14, 48, 49]
Werner Syndrome	Doxorubicin + Cisplatin	Poor chemotherapy tolerance, cardiovascular risks	RECQL4 inhibitors, Pembrolizumab	[50, 51]
Bloom Syndrome	Anthracyclines + Cisplatin	High toxicity to DNA-damaging drugs	RecQ helicase-targeted therapies	[40, 41]

The treatment of RTS-related osteosarcoma faces dual challenges: patients’ poor tolerance to the toxicity of conventional chemotherapy (e.g., increased risks of myelosuppression and mucositis) [44] and the more aggressive biological behavior of the tumors. Clinical data indicate that, despite the use of doxorubicin-based EURAMOS-1/COSS protocols, the 5-year survival rate for RTS patients remains lower than that for sporadic osteosarcoma (approximately 50–60% vs. 70%) [15]. Surgical resection remains the cornerstone of local control, but the balance between wide excision and limb preservation is critical. In recent years, targeted therapies (e.g., cabozantinib and pazopanib) have shown potential in suppressing tumor growth in RECQL4-deficient models, though their clinical efficacy requires further validation [45]. Given the elevated risk of secondary malignancies (e.g., breast cancer, skin cancer) in RTS patients, multidisciplinary follow-up, including regular imaging screening and genetic counseling, is recommended post-osteosarcoma treatment [46]. Early genetic diagnosis and personalized treatment strategies are key to improving prognosis.

### Li-Fraumeni syndrome and osteosarcoma

#### Molecular mechanisms of TP53 mutations (Table 2)

The TP53 gene, a critical tumor suppressor, plays a central role in Li-Fraumeni syndrome (LFS)-related osteosarcoma [31]. The p53 protein, encoded by TP53, maintains genomic stability by regulating cell cycle arrest, apoptosis, and DNA repair [52]. Germline TP53 mutations in LFS patients (e.g., missense, nonsense, or splice-site mutations) result in loss of p53 function, leading to genomic instability and tumorigenesis [32]. Studies show that TP53 mutation frequency is significantly higher in osteosarcoma than in other sarcoma subtypes, often accompanied by loss of heterozygosity (i.e., the “second hit” model) [53]. Additionally, TP53 mutations promote osteosarcoma cell invasion by activating the MEK-ERK pathway and synergize with mutations in NOTCH1 and FOS to drive tumor progression [54]. Notably, certain TP53 mutations (e.g., p.Arg267Trp) may exacerbate phenotypic heterogeneity by disrupting interactions with CCAR2, impairing p53’s transcriptional regulatory functions [55]. Of particular relevance in certain populations, such as in Southern Brazil, is the founder *TP53* R337H mutation. This specific variant is strongly associated with a very high incidence of pediatric adrenocortical carcinoma and a significantly increased risk of childhood osteosarcoma [56].

Osteosarcoma is characterized by profound genomic instability, which contributes to its aggressive behavior and evolution. This instability is frequently driven by catastrophic events such as chromothripsis, a process wherein dozens to hundreds of clustered genomic rearrangements occur in a single catastrophic episode. Critically, seminal studies have established that loss of TP53 function is a fundamental prerequisite for cells to undergo and survive chromothripsis [57, 58]. The underlying mechanism involves the survival of cells with mitotic errors (e.g., micronuclei formation) that, in a TP53-wildtype context, would be eliminated by apoptosis [57]. In TP53-deficient cells, however, the encapsulated chromosomal DNA within micronuclei undergoes pulverization and error-prone repair, leading to the complex rearrangements hallmark of chromothripsis. In osteosarcoma, where TP53 pathway inactivation is near-universal, this mechanism provides a rapid ‘evolutionary leap’ by simultaneously amplifying oncogenes and deleting tumor suppressors, thereby fueling tumor progression and heterogeneity [53].

#### Clinical characteristics and familial inheritance patterns (Table 1)

LFS-related osteosarcoma is characterized by early onset and familial clustering, with approximately 28% of osteosarcoma patients carrying highly penetrant cancer susceptibility gene variants, predominantly TP53 mutations [1]. Clinically, it manifests in childhood or adolescence (median diagnosis age of 16 years) and is often accompanied by other LFS-associated tumors (e.g., breast cancer, brain tumors) [3]. The inheritance pattern is autosomal dominant, but phenotypic heterogeneity is pronounced, with family members potentially presenting different tumor types or ages of onset [59]. Case reports indicate that some patients present with osteosarcoma as the initial manifestation, with LFS diagnosed only after subsequent malignancies (e.g., breast cancer, glioma) [60]. Moreover, LFS patients have a significantly elevated risk of second primary malignancies; for example, a study reported a 17-year-old osteosarcoma survivor developing HER2-positive breast cancer 10 years later, with genetic testing confirming a TP53 germline mutation [61].

#### Treatment strategies and monitoring (Table 3)

The treatment of LFS-related osteosarcoma must balance tumor eradication with minimizing the risk of secondary cancers [9]. Current standard protocols involve surgery combined with anthracycline-based chemotherapy (e.g., doxorubicin). Radiotherapy is absolutely contraindicated in patients with confirmed or clinically suspected Li-Fraumeni syndrome. This is due to the profoundly elevated risk of radiation-induced secondary malignancies, which is a hallmark of *TP53*-deficient cells. Functional restoration strategies targeting TP53 mutations (e.g., p53 activators) are under investigation, while targeted therapies (e.g., cabozantinib, sorafenib) have shown promise in clinical trials [47]. Monitoring involves a multidisciplinary follow-up approach, including whole-body MRI, 68 Ga-DOTATATE PET/CT, and cascade screening for TP53 mutation carriers within families [62]. International guidelines recommend lifelong annual screening for LFS patients, covering high-risk organs such as the breast, brain, and hematopoietic system. Genetic counseling is critical, particularly for young patients or those with a family history of multiple primary cancers, to assess the need for germline testing and develop individualized risk management plans.

### Secondary osteosarcoma in retinoblastoma survivors

#### RB1 gene mutations and secondary tumor risk (Table 2)

The risk of secondary osteosarcoma in retinoblastoma (Rb) survivors is closely linked to germline mutations in the RB1 gene [34]. Studies show that patients with RB1 mutations are not only susceptible to retinoblastoma but also have a significantly elevated risk of osteosarcoma. The RB1 gene encodes the retinoblastoma protein (pRB), a key regulator of the cell cycle, and its loss leads to genomic instability and tumor predisposition [63]. In vitro studies demonstrate that mesenchymal stem cells with heterozygous RB1 mutations exhibit reduced RB1 expression, alongside altered expression of other tumor suppressors (e.g., TP53) [35]. Additionally, RB1 mutations may promote osteosarcoma development by enhancing the activity of oncogenes such as MET [45]. These findings underscore the central role of RB1 in osteosarcoma pathogenesis and provide a foundation for genetic counseling.

pRB directly binds to the master osteogenic transcription factor RUNX2 via its pocket domain, and this interaction is critical for recruiting RUNX2 to target gene promoters and activating the transcriptional program essential for terminal osteogenic differentiation [64, 65]. Loss of RB1 function disrupts this pRB-RUNX2 complex, leading to dysregulated RUNX2 activity. The consequence is not a simple loss of RUNX2 function but a differentiation block: osteoprogenitor cells enter the osteoblastic lineage but fail to exit the cell cycle and complete maturation. These proliferating, undifferentiated cells accumulate, particularly in areas of active bone growth such as the metaphyses of long bones, ultimately leading to tumor formation [66, 67]. Therefore, the disruption of the pRB-RUNX2 interaction and the resulting differentiation block constitute a key tissue-specific mechanism linking the general cell cycle defect caused by RB1 loss to the development of osteosarcoma.

#### Radiotherapy and secondary osteosarcoma (Table 1)

Radiotherapy, a key treatment for retinoblastoma, is a well-established risk factor for secondary osteosarcoma [68]. Studies indicate that Rb patients receiving external beam radiotherapy have a 35-year cumulative incidence of secondary osteosarcoma of up to 32.6%, predominantly in craniofacial bones (e.g., mandible, skull base) within the radiation field [20, 69]. The mechanisms underlying radiotherapy-induced osteosarcoma include direct DNA damage, increased genomic instability, and alterations in the local microenvironment [70]. Notably, neither age at radiotherapy (e.g., < 12 months) nor bilateral irradiation significantly increases the risk, but radiation dose and field size correlate closely with the latency period (median 16.9 years) [71]. Clinically, radiotherapy-induced osteosarcomas often exhibit aggressive growth and may be associated with secondary RB1 mutations [21], suggesting a synergistic role of radiotherapy and genetic predisposition in tumorigenesis.

#### Clinical management and prevention (Table 3)

Long-term management of Rb survivors requires a multidisciplinary approach to minimize the risk of secondary osteosarcoma. Radiotherapy should be avoided in hereditary Rb patients, particularly infants, with chemotherapy or local treatments (e.g., brachytherapy) prioritized [48]. For patients who have received radiotherapy, lifelong follow-up is recommended, including annual clinical evaluations and imaging surveillance (e.g., Tc-99 m bone scans or MRI) to detect asymptomatic lesions early [72]. Studies show that early osteosarcomas detected via routine bone scans have a 5-year survival rate of up to 86%, significantly higher than cases diagnosed after symptom onset [49]. Additionally, RB1 gene testing is recommended for all Rb patients, with mutation carriers offered genetic counseling, including family screening and individualized tumor risk assessments. For patients developing osteosarcoma, treatment mirrors that of primary osteosarcoma (neoadjuvant chemotherapy + wide resection + adjuvant chemotherapy), but repeated radiotherapy exposure should be avoided to reduce the risk of secondary malignancies.

### Werner syndrome and osteosarcoma

#### WRN gene mutations and premature aging features (Table 2)

Werner syndrome (WS), a rare autosomal recessive disorder, is caused by mutations in the WRN gene (located at 8p12), which encodes a member of the RecQ DNA helicase family [73]. The WRN protein plays a critical role in DNA repair, telomere maintenance, and genomic stability [37]. Mutations lead to loss of WRN protein function, resulting in premature aging features, including cataracts, skin sclerosis, osteoporosis, diabetes, and atherosclerosis appearing after adolescence [38]. Notably, WS patients have a significantly elevated cancer risk, particularly for soft tissue sarcomas and osteosarcoma, directly linked to genomic instability caused by DNA repair defects [16]. Studies suggest that WRN loss accelerates cellular senescence and promotes tumorigenesis, potentially through telomere dysfunction and mitochondrial metabolic abnormalities [74]. Additionally, WRN mutations may enhance osteosarcoma susceptibility by affecting p53 pathways and homologous recombination repair [39].

#### Clinical features of osteosarcoma (Table 1)

Osteosarcoma in WS patients exhibits distinct clinical and molecular characteristics. Compared to sporadic osteosarcoma, WS-related osteosarcoma has a later onset (typically 30–50 years) [22] and frequently affects sites other than long bones, such as the pelvis or spine [23]. At the molecular level, these tumors frequently harbor secondary mutations in tumor suppressor genes (e.g., TP53, RB1), which synergize with WRN defects to drive malignant transformation [45]. Genomic analyses also reveal high-frequency copy number variations (e.g., MYC amplification) and driver mutations in NOTCH1 and FOS [54]. Furthermore, CD133+ tumor stem cells are more active in WS patients, potentially contributing to increased invasiveness and drug resistance [75]. These features pose significant challenges for the diagnosis and treatment of WS-related osteosarcoma.

#### Treatment challenges and prognosis (Table 3)

The treatment of WS-associated osteosarcoma is complicated by patients’ systemic conditions and molecular defects. Cardiovascular risk is the core manifestation of the premature aging phenotype of Werner syndrome, which is characterized by extensive, premature, and dangerous accelerated atherosclerosis, especially coronary artery disease, and characteristic severe peripheral artery disease. Therefore, Werner syndrome with osteosarcoma is poorly tolerated by chemotherapy. [50]. Moreover, WRN defects impair DNA repair, potentially reducing radiotherapy efficacy while elevating the risk of secondary malignancies [70]. Clinical data indicate that the 5-year survival rate for WS patients with osteosarcoma is less than 30%, significantly lower than that for sporadic osteosarcoma (approximately 60–70%) [51]. To optimize prognosis, individualized treatment plans and multidisciplinary follow-up (including imaging and genetic counseling) are recommended to monitor secondary tumor risks.

### Bloom syndrome and osteosarcoma

#### BLM gene mutations and molecular mechanisms (Table 2)

Bloom syndrome (BS) is an autosomal recessive disorder caused by mutations in the BLM gene, which encodes a member of the RecQ helicase family critical for maintaining genomic stability [40]. BLM mutations lead to increased genomic instability and significantly elevated cancer susceptibility [41]. The BLM protein, a DNA helicase, is involved in DNA replication, recombination, and repair, particularly in homologous recombination repair (HRR) pathways [76]. BLM defects result in an inability to dissolve Holliday junctions, thereby increasing the incidence of chromosome breaks and sister chromatid exchanges [42]. In osteosarcoma, BLM mutations may promote oncogenic mutation accumulation by disrupting DNA damage response mechanisms [16]. Additionally, mutations in other RecQ helicase family members (e.g., WRN, RECQL4) are also associated with osteosarcoma, highlighting the family’s critical role in tumorigenesis [39].

#### Clinical characteristics and diagnosis (Table 1)

Patients with Bloom syndrome-related osteosarcoma typically exhibit BS’s hallmark clinical features, including short stature, photosensitive rashes, immunodeficiency, and characteristic facial morphology (e.g., narrow face, small mandible) [24]. These patients develop osteosarcoma at a younger age and may have concurrent malignancies (e.g., leukemia, lymphoma) [1]. Diagnosis relies on conventional imaging and histopathological examinations, with genetic testing to confirm BLM gene and biallelic mutations being essential for BS confirmation [77]. Notably, some patients are initially misdiagnosed with other RecQ helicase-related syndromes (e.g., Rothmund-Thomson syndrome), underscoring the importance of genetic testing for differential diagnosis [19].

## Conclusion

Syndrome-related osteosarcomas, due to their unique clinical characteristics and molecular mechanisms, represent a critical focus in osteosarcoma research. These tumors are closely associated with hereditary syndromes such as Li-Fraumeni syndrome and hereditary retinoblastoma syndrome and others, with pathogenesis involving complex gene mutations and signaling pathway aberrations. Early diagnosis and personalized treatment are pivotal for improving patient outcomes. Although studies have confirmed the central role of TP53, RB1, and other gene mutations in syndrome-related osteosarcomas, discrepancies exist across studies regarding the pathogenicity of specific genes. Future research should integrate multi-omics data with clinical phenotypes to more precisely elucidate the pathogenic mechanisms of these genes.

Therapeutically, the efficacy of conventional chemotherapy and surgery is limited, while targeted therapies and immunotherapies remain in exploratory stages. Inhibitors targeting specific signaling pathways (e.g., mTOR, CDK4/6) may be effective, but patient responses vary significantly. Additionally, genetic counseling and regular monitoring are crucial for reducing incidence and improving early detection rates in high-risk populations. However, optimizing surveillance protocols requires further evidence-based support. Through multidisciplinary collaboration (e.g., genetics, oncology, imaging), combined with precision medicine and personalized treatment strategies, significant improvements in patient survival and quality of life are anticipated in the future [78].

## Data Availability

Data sharing not applicable as no datasets were generated.

## Abbreviations

BS: Bloom Syndrome
COSS: Cooperative Osteosarcoma Study
EURAMOS-1: European and American Osteosarcoma Study Group 1
HRR: Homologous Recombination Repair
LFS: Li-Fraumeni Syndrome
MAPK: Mitogen-Activated Protein Kinase
OS: Osteosarcoma
PET/CT: Positron Emission Tomography/Computed Tomography
PI3K/Akt: Phosphatidylinositol 3-Kinase/Protein Kinase B
pRB: Retinoblastoma Protein
Rb: Retinoblastoma
RTS: Rothmund-Thomson Syndrome
WS: Werner Syndrome

## References

[1] Mirabello L, Zhu B, Koster R, et al. Frequency of pathogenic germline variants in cancer-susceptibility genes in patients with osteosarcoma. JAMA Oncol. 2020;6(5):724–34. 32191290 10.1001/jamaoncol.2020.0197 PMC7082769

[2] Gianferante DM, Mirabello L, Savage SA. Germline and somatic genetics of osteosarcoma - connecting aetiology, biology and therapy. Nat Rev Endocrinol. 2017;13(8):480–91. 28338660 10.1038/nrendo.2017.16

[3] Mai PL, Best AF, Peters JA, et al. Risks of first and subsequent cancers among TP53 mutation carriers in the national cancer institute Li-Fraumeni syndrome cohort. Cancer. 2016;122(23):3673–81. 27496084 10.1002/cncr.30248 PMC5115949

[4] Wu J, Lu C, Wang Z, et al. Immuno-genomic landscape of osteosarcoma. Nat Commun. 2020;11(1):1008. 32081846 10.1038/s41467-020-14646-w PMC7035358

[5] Hickson ID, Wu Z. RECQL4 helicase in DNA repair and cancer. Biochem Soc Trans. 2020;48(6):2399–408.33196096

[6] Sekar D, Mani P, Biruntha M, et al. Dissecting the role of miR-21 in different types of cancer. Pathol Res Pract. 2019;215(12):152697.31704155

[7] Ding Y, Chen Q. Wnt/β-catenin signaling pathway: an attractive potential therapeutic target in osteosarcoma. Front Oncol. 2025;14:1456959. 40028002 10.3389/fonc.2024.1456959 PMC11867957

[8] Liau JY, Tsai JH, Yang CY, et al. Alternative lengthening of telomeres phenotype in malignant vascular tumors is highly associated with loss of ATRX expression and is frequently observed in hepatic angiosarcomas. Hum Pathol. 2015;46(9):1360–66. 26190196 10.1016/j.humpath.2015.05.019

[9] Özer Ö, Hickson ID. Pathways for maintenance of telomeres and common fragile sites during DNA replication stress. Open Biol. 2018;8(4):180018. 29695617 10.1098/rsob.180018 PMC5936717

[10] Ghosh AK, Rossi ML, Singh DK, et al. RECQL4, the protein mutated in Rothmund-Thomson syndrome, functions in telomere maintenance. J Biol Chem. 2012;287(1):196–209. 22039056 10.1074/jbc.M111.295063 PMC3249070

[11] Wang LL, Gannavarapu A, Kozinetz CA, et al. Association between osteosarcoma and deleterious mutations in the RECQL4 gene in Rothmund-Thomson syndrome. J Natl Cancer Inst. 2003;95(9):669–74. 12734318 10.1093/jnci/95.9.669

[12] He H, Ni J, Huang J. Molecular mechanisms of chemoresistance in osteosarcoma. Oncol Lett. 2018;16(4):4083–92. 10.3892/ol.2014.1935 PMC3997672 24765137

[13] Frebourg T, Bajalica Lagercrantz S, Oliveira C, et al. Guidelines for the Li-Fraumeni and heritable TP53-related cancer syndromes. Eur J Hum Genet. 2020;28(10):1379–86. 32457520 10.1038/s41431-020-0638-4 PMC7609280

[14] Yu S, Yao X. Advances on immunotherapy for osteosarcoma. Mol Cancer. 2024;23(1):192. 39245737 10.1186/s12943-024-02105-9 PMC11382402

[15] Larizza L, Roversi G, Volpi L. Rothmund-Thomson syndrome. Orphanet J Rare Dis. 2010;5:2. 20113479 10.1186/1750-1172-5-2 PMC2826297

[16] Lu L, Jin W, Wang LL. RECQ DNA helicases and osteosarcoma. Adv Exp Med Biol. 2020;1258:37–54. 32767233 10.1007/978-3-030-43085-6_3

[17] Simon T, Kohlhase J, Wilhelm C, et al. Multiple malignant diseases in a patient with Rothmund-Thomson syndrome with RECQL4 mutations: case report and literature review. Am J Med Genet A. 2010;152A(6):1575–79. 20503338 10.1002/ajmg.a.33427

[18] Salih A, Inoue S, Onwuzurike N. Rothmund-Thomson syndrome (RTS) with osteosarcoma due to RECQL4 mutation. BMJ Case Rep. 2018. 2018;bcr2017222384. 10.1136/bcr-2017-222384 PMC5786930 29367366

[19] Martins DJ, Di Lazzaro Filho R, Bertola DR, Hoch NC. Rothmund-Thomson Syndrome, a disorder far from solved. Front Aging. 2023;4:1296409. 38021400 10.3389/fragi.2023.1296409 PMC10676203

[20] Schonfeld SJ, Kleinerman RA, Abramson DH, et al. Late effects in survivors of high-risk retinoblastoma: second primary cancers. Pediatr Blood Cancer. 2021;68(10):e29206.

[21] da Silva Amp, Cunha Amaral D, Carneiro Filho LF, et al. Risk factors for secondary neoplasms in retinoblastoma survivors: a systematic literature review. Expert Rev Anticancer Ther. 2025;25(10):1195–202. 40632082 10.1080/14737140.2025.2532110

[22] Goto M, Miller RW, Ishikawa Y, Sugano H. Excess of rare cancers in Werner syndrome (adult progeria). Cancer Epidemiol Biomarker Prev. 1996;5(4):239–46. 8722214

[23] Ishikawa Y, Miller RW, Machinami R, Sugano H, Goto M. Atypical osteosarcomas in Werner syndrome (adult progeria). Jpn J Cancer Res. 2000;91(12):1345–49. 11123436 10.1111/j.1349-7006.2000.tb00924.x PMC5926298

[24] Cunniff C, Djavid AR, Carrubba S, et al. Health supervision for people with Bloom syndrome. Am J Med Genet A. 2018;176(9):1872–81. 30055079 10.1002/ajmg.a.40374

[25] Kitao S, Shimamoto A, Goto M, et al. Mutations in RECQL4 cause a subset of cases of Rothmund-Thomson syndrome. Nat Genet. 1999;22(1):82–84. 10319867 10.1038/8788

[26] Singh DK, Karmakar P, Aamann M, et al. The involvement of human RECQL4 in DNA double-strand break repair. Aging Cell. 2010;9(3):358–71. 20222902 10.1111/j.1474-9726.2010.00562.x PMC4624395

[27] Maciaszek JL, Oak N, Chen W, et al. Enrichment of heterozygous germline RECQL4 loss-of-function variants in pediatric osteosarcoma. Cold Spring Harb Mol Case Stud. 2019;5(5):a004218. 10.1101/mcs.a004218 PMC6824257 31604778

[28] Lu L, Jin W, Wang LL. Aging in Rothmund-Thomson syndrome and related RECQL4 genetic disorders. Ageing Res Rev. 2017;33:30–35. 27287744 10.1016/j.arr.2016.06.002

[29] Morrow JJ, Khanna C. Osteosarcoma genetics and epigenetics: emerging biology and candidate therapies. Crit Rev Oncog. 2015;20(3–4):173–97. 26349415 10.1615/critrevoncog.2015013713 PMC4894524

[30] Ng AJM, Walia MK, Smeets MF, et al. The DNA helicase recql4 is required for normal osteoblast expansion and osteosarcoma formation. PLoS Genet. 2015;11(4):e1005160. 10.1371/journal.pgen.1005160 PMC4393104 25859855

[31] Malkin D. Li-Fraumeni syndrome. Genes Cancer. 2011;2(4):475–84. 21779515 10.1177/1947601911413466 PMC3135649

[32] Guha T, Malkin D. Inherited TP53 mutations and the Li-Fraumeni syndrome. Cold Spring Harb Perspect Med. 2017;7(4):a026187. 10.1101/cshperspect.a026187 PMC5378014 28270529

[33] Kratz CP, Achatz MI, Brugières L, et al. Cancer screening recommendations for individuals with Li-Fraumeni syndrome. Clin Cancer Res. 2017;23(11):e38–45. 10.1158/1078-0432.CCR-17-0408 28572266

[34] Kleinerman RA, Tucker MA, Tarone RE, et al. Risk of new cancers after radiotherapy in long-term survivors of retinoblastoma: an extended follow-up. J Clin Oncol. 2005;23(10):2272–79. 15800318 10.1200/JCO.2005.05.054

[35] Zhang J, Walsh MF, Wu G, et al. Germline mutations in predisposition genes in pediatric cancer. N Engl J Med. 2015;373(24):2336–46. 26580448 10.1056/NEJMoa1508054 PMC4734119

[36] Wong JR, Morton LM, Tucker MA, et al. Risk of subsequent malignant neoplasms in long-term hereditary retinoblastoma survivors after chemotherapy and radiotherapy. J Clin Oncol. 2014;32(29):3284–90. 25185089 10.1200/JCO.2013.54.7844 PMC4178525

[37] Shamanna RA, Croteau DL, Lee JH, Bohr VA. The Werner syndrome protein is involved in RNA polymerase II transcription. Mol Biol Cell. 1999;10(8):2655–68. 10436020 10.1091/mbc.10.8.2655 PMC25497

[38] Lauper JM, Krause A, Vaughan TL, Monnat RJ Jr. Spectrum and risk of neoplasia in Werner syndrome: a systematic review. PLoS One. 2013;8(4):e59709. 10.1371/journal.pone.0059709 PMC3613408 23573208

[39] Mao FJ, Sidorova JM, Lauper JM, et al. The human WRN and BLM RecQ helicases differentially regulate cell proliferation and survival after chemotherapeutic DNA damage. Cancer Res. 2020;70(16):6548–55. 10.1158/0008-5472.CAN-10-0475 PMC2941797 20663905

[40] Ellis NA, German J. Molecular genetics of Bloom’s syndrome. Hum Mol Genet. 1996, 5 Spec: No, 1457-1463. 10.1093/hmg/5.supplement_1.1457 8875252

[41] Flanagan M, Cunniff CM Bloom syndrome. in: Pagon RA, Adam MP, Ardinger HH, et al, editors. GeneReviews®. Seattle: University of Washington; 2023.

[42] Amor-Guéret M. Bloom syndrome, genomic instability and cancer: the SOS-like hypothesis. Cancer Lett. 2006;236(1):1–12. 15950375 10.1016/j.canlet.2005.04.023

[43] German J. Bloom’s syndrome. XX. The first 100 cancers. Cancer Genet Cytogenet. 1997;93(1):100–06. 9062585 10.1016/s0165-4608(96)00336-6

[44] Hicks MJ, Roth JR, Kozinetz CA, Wang LL. Clinicopathologic features of osteosarcoma in patients with Rothmund-Thomson syndrome. J Clin Oncol. 2007;25(4):370–75. 17264332 10.1200/JCO.2006.08.4558

[45] Pekarek L, De la Torre-Escuredo B, Fraile-Martinez O, et al. Towards the search for potential biomarkers in osteosarcoma: state-of-the-art and translational expectations. Int J Mol Sci. 2022;23(23):14939. 36499267 10.3390/ijms232314939 PMC9740676

[46] Greer MC. Imaging of cancer predisposition syndromes. Pediatr Radiol. 2018;48(9):1364–75. 30078044 10.1007/s00247-018-4113-0

[47] Joerger AC, Fersht AR. The p53 pathway: origins, inactivation in Cancer, and emerging therapeutic approaches. Annu Rev Biochem. 2016;85:375–404. 27145840 10.1146/annurev-biochem-060815-014710

[48] Abramson DH, Shields CL, Munier FL, et al. Treatment of retinoblastoma in 2015: agreement and disagreement. JAMA Ophthalmol. 2015;133(11):1341–47. 26378747 10.1001/jamaophthalmol.2015.3108

[49] Chai P, Luo Y, Yu J, et al. Clinical characteristics and germline mutation spectrum of RB1 in Chinese patients with retinoblastoma: a dual-center study of 145 patients. Exp Eye Res. 2021;205:108456. 33493472 10.1016/j.exer.2021.108456

[50] Hisama FM, Kubisch C, Martin GM, Oshima J. Clinical utility gene card for: werner syndrome. Eur J Hum Genet. 2012;20(5):497. 10.1038/ejhg.2011.265 PMC3330230 22258520

[51] Khraishi M, Howard B, Little H. A patient with Werner’s syndrome and osteosarcoma presenting as scleroderma. J Rheumatol. 1992;19(5):810–13. 1613716

[52] Kastenhuber ER, Lowe SW. Putting p53 in context. Cell. 2017;170(6):1062–78. 28886379 10.1016/j.cell.2017.08.028 PMC5743327

[53] Chen X, Bahrami A, Pappo A, et al. Recurrent somatic structural variations contribute to tumorigenesis in pediatric osteosarcoma. Cell Rep. 2014;7(1):104–12. 24703847 10.1016/j.celrep.2014.03.003 PMC4096827

[54] Perry JA, Kiezun A, Tonzi P, et al. Complementary genomic approaches highlight the PI3K/mTOR pathway as a common vulnerability in osteosarcoma. Proc Natl Acad Sci U S A. 2014;111(51):E5564–73. 10.1073/pnas.1419260111 PMC4280630 25512523

[55] Iyer DR, Harada N, Clairmont C, et al. CCAR2 functions downstream of the shieldin complex to promote double-strand break end-joining. Proc Natl Acad Sci U S A. 2022;119(49):e2214935119. 10.1073/pnas.2214935119 PMC9894118 36442094

[56] Giacomazzi J, Selistre S, Duarte J, et al. TP53 p.R337H is a conditional cancer-predisposing mutation: further evidence from a homozygous patient. BMC Cancer. 2013;13:187. 23570263 10.1186/1471-2407-13-187 PMC3637265

[57] Cortés-Ciriano I, Lee JJ, Xi R, et al. Comprehensive analysis of chromothripsis in 2, 658 human cancers using whole-genome sequencing. Nat Genet. 2020;52(3):331–41. 32025003 10.1038/s41588-019-0576-7 PMC7058534

[58] Bakhoum SF, Ngo B, Laughney AM, et al. Chromosomal instability drives metastasis through a cytosolic DNA response. Nature. 2018;553(7689):467–72. 29342134 10.1038/nature25432 PMC5785464

[59] Kratz CP, Achatz MI, Brugières L, et al. Cancer screening recommendations for individuals with Li-Fraumeni syndrome. Clin Cancer Res. 2017;23(13):e38–45. 10.1158/1078-0432.CCR-17-0408 28572266

[60] Ballinger ML, Goode DL, Ray-Coquard I, et al. Monogenic and polygenic determinants of sarcoma risk: an international genetic study. Lancet Oncol. 2016;17(9):1261–71. 27498913 10.1016/S1470-2045(16)30147-4

[61] Forbes Shepherd R, Keogh LA, Werner-Lin A, Delatycki MB, Forrest LE. Benefits and burdens of risk management for young people with inherited cancer: a focus on Li-Fraumeni syndrome. Aust J Gen Pract. 2021;50(8):538–44. 34333565 10.31128/AJGP-04-21-5954

[62] Villani A, Shore A, Wasserman JD, et al. Biochemical and imaging surveillance in germline TP53 mutation carriers with Li-Fraumeni syndrome: 11-year follow-up of a prospective observational study. Lancet Oncol. 2016;17(9):1295–305. 27501770 10.1016/S1470-2045(16)30249-2

[63] Dyson NJ. RB1: a prototype tumor suppressor and an enigma. Genes Dev. 2016;30(13):1492–502. 27401552 10.1101/gad.282145.116 PMC4949322

[64] Thomas DM, Carty SA, Piscopo DM, et al. The retinoblastoma protein acts as a transcriptional coactivator required for osteogenic differentiation. Mol Cell. 2001;8(2):303–16. 11545733 10.1016/s1097-2765(01)00327-6

[65] Giacinti C, Giordano A. RB and cell cycle progression. Oncogene. 2006;25(38):5220–27. 16936740 10.1038/sj.onc.1209615

[66] Calo E, Quintero-Estades JA, Danielian PS, Nedelcu S, Berman SD, Lees JA. Rb regulates fate choice and lineage commitment in vivo. Nature. 2010;466(7310):1110–14. 20686481 10.1038/nature09264 PMC2933655

[67] Walkley CR, Shea JM, Sims NA, Purton LE, Orkin SH. Rb regulates interactions between hematopoietic stem cells and their bone marrow microenvironment. Cell. 2007;129(6):1081–95. 17574022 10.1016/j.cell.2007.03.055 PMC2768301

[68] Kleinerman RA, Schonfeld SJ, Tucker MA. Sarcomas in hereditary retinoblastoma. Clin Sarcoma Res. 2012;2(1):15. 23036192 10.1186/2045-3329-2-15 PMC3499233

[69] Sethi RV, Shih HA, Yeap BY, et al. Second nonocular tumors among survivors of retinoblastoma treated with contemporary photon and proton radiotherapy. Cancer. 2014;120(1):126–33. 24122173 10.1002/cncr.28387 PMC3959122

[70] Casey DL, Vogelius IR, Brodin NP, et al. Risk of subsequent neoplasms in childhood Cancer survivors after radiation therapy: a PENTEC Comprehensive review. Int J Radiat Oncol Biol Phys. 2024;119(2):640–54. 37777927 10.1016/j.ijrobp.2023.07.025

[71] Temming P, Arendt M, Viehmann A, et al. Incidence of second cancers after radiotherapy and systemic chemotherapy in hereditary retinoblastoma survivors: a report from the German reference center. Pediatr Blood Cancer. 2017;64(1):71–80. 27567086 10.1002/pbc.26193

[72] de Jong MC, Kors WA, de Graaf P, et al. The incidence of trilateral retinoblastoma: a systematic review and meta-analysis. Am J Ophthalmol. 2015;160(6):1116–26. 26374932 10.1016/j.ajo.2015.09.009

[73] Oshima J, Sidorova JM, Monnat RJ Jr. Werner syndrome: clinical features, pathogenesis and potential therapeutic interventions. Ageing Res Rev. 2017;33:105–14. 26993153 10.1016/j.arr.2016.03.002 PMC5025328

[74] Saha B, Zitnik G, Johnson S, et al. DNA damage accumulation and TRF2 degradation in atypical Werner syndrome fibroblasts with LMNA mutations. Front Genet. 2013;4:129. 23847654 10.3389/fgene.2013.00129 PMC3701863

[75] Beird HC, Bielack SS, Flanagan AM, et al. Osteosarcoma. Nat Rev Dis Primers. 2022;8(1):77. 36481668 10.1038/s41572-022-00409-y

[76] Blackford AN, Jackson SP. ATM, ATR, and DNA-PK: the trinity at the heart of the DNA damage response. Mol Cell. 2017;66(6):801–17. 28622525 10.1016/j.molcel.2017.05.015

[77] Pacaud C, Nazon C, Pages M, et al. Management of paediatric cancers associated with Bloom syndrome. Hum Mutat. 2025. 2025;7065233. 10.1155/humu/7065233 PMC12245492 40641635

[78] Strauss SJ, Frezza AM, Abecassis N, et al. Bone sarcomas: ESMO-EURACAN-GENTURIS-ERN PaedCan clinical practice guideline for diagnosis, treatment and follow-up. Ann Oncol. 2021;32(12):1520–36. 34500044 10.1016/j.annonc.2021.08.1995

